# Structural Characterization and Physicochemical Properties of Functionally Porous Proton-Exchange Membrane Based on PVDF-SPA Graft Copolymers

**DOI:** 10.3390/ijms25010598

**Published:** 2024-01-02

**Authors:** Maria Ponomar, Valentina Ruleva, Veronika Sarapulova, Natalia Pismenskaya, Victor Nikonenko, Alina Maryasevskaya, Denis Anokhin, Dimitri Ivanov, Jeet Sharma, Vaibhav Kulshrestha, Bruno Améduri

**Affiliations:** 1 Department of Physical Chemistry, Kuban State University, 350040 Krasnodar, Russia; 2 Faculty of Fundamental Physical and Chemical Engineering, Lomonosov Moscow State University, 119991 Moscow, Russiabruno.ameduri@enscm.fr (B.A.); 3 Federal Research Center of Problems of Chemical Physics and Medicinal Chemistry Russian Academy of Sciences, 142432 Chernogolovka, Russia; 4 Center for Genetics and Life Science, Sirius University of Science and Technology, 354340 Sochi, Russia; 5 Institut de Sciences des Matériaux de Mulhouse-IS2M, CNRS UMR 7361, 68057 Mulhouse, France; 6 Institute Charles Gerhardt, CNRS, University of Montpellier, Ecole Nationale Supérieure de Chimie de Montpellier, 34000 Montpellier, France; jeet2412s@gmail.com; 7 Membrane Science and Separation Technology Division, Council of Scientific and Industrial Research, Central Salt and Marine Chemicals Research Institute (CSIR-CSMCRI), Bhavnagar 364002, India; 8 Academy of Scientific and Innovative Research (AcSIR), Ghaziabad 201002, India

**Keywords:** conductivity, diffusion permeability, energy production and storage, ion transport, proton-exchange membranes, fuel cell, graft copolymer, electrochemical characterization, porous structure

## Abstract

Fluorinated proton-exchange membranes (PEMs) based on graft copolymers of dehydrofluorinated polyvinylidene fluoride (D-PVDF), 3-sulfopropyl acrylate (SPA), and 1H, 1H, 2H-perfluoro-1-hexene (PFH) were prepared via free radical copolymerization and characterized for fuel cell application. The membrane morphology and physical properties were studied via small-(SAXS) and wide-angle X-ray scattering (WAXS), SEM, and DSC. It was found that the crystallinity degree is 17% for PEM-RCF (co-polymer with SPA) and 16% for PEM-RCF-2 (copolymer with SPA and PFH). The designed membranes possess crystallite grains of 5–6 nm in diameter. SEM images reveal a structure with open pores on the surface of diameters from 20 to 140 nm. Their transport and electrochemical characterization shows that the lowest membrane area resistance (0.9 Ωcm^2^) is comparable to perfluorosulfonic acid PEMs (such as Nafion^®^) and polyvinylidene fluoride (PVDF) based CJMC cation-exchange membranes (ChemJoy Polymer Materials, China). Key transport and physicochemical properties of new and commercial membranes were compared. The PEM-RCF permeability to NaCl diffusion is rather high, which is due to a relatively low concentration of fixed sulfonate groups. Voltammetry confers that the electrochemical behavior of new PEM correlates to that of commercial cation-exchange membranes, while the ionic conductivity reveals an impact of the extended pores, as in track-etched membranes.

## 1. Introduction

Sustainable development of the modern society is closely connected to efficient and safe production and the usage of energy. Mankind is constantly searching for new clean sources of energy based on concepts that are ecologically and economically appropriate for the long-term future. Polymer electrolyte membrane fuel cells (PEMFCs) can generate electricity using hydrogen (or other fuel). Polymer electrolyte membrane water electrolyzers (PEMWEs) can generate hydrogen. Coupled together, PEMFCs and PEMWEs can form the basis of an efficient green hydrogen zero-carbon technology for energy production and storage [1,2,3]. It is noted that the main features of such systems enlist low operating temperature, high energy-conversion efficiency, low emission, quiet operation, and easy scale up [1,4]. The proton exchange membrane is a major component in these devices and channelizes the conduction of protons from the anode to the cathode.

Numerous studies and review papers have been devoted to the study of PEM [1,5,6,7,8,9,10,11,12] and suggest that the characteristics of the PEM has a significant effect on the performance and durability of the PEMFCs. Among all commercial samples for the PEMFC applications, the most used membranes are perfluorosulfonic acid (PFSA) due to its stability, long-term performance, mechanical strength, and low conduction resistance [13,14]. Standard reference PFSA used in energy conversion systems is the Nafion^®^ membrane, developed by the DuPont Company (Wilmington, Delaware, United States), which consists of an aliphatic perfluorinated backbone with ether-linked side chains bearing sulfonated cation exchange sites. However, despite all the advantages of the Nafion-like PFSA membranes, there are several challenges viz dehydration-induced inoperability at high temperatures, gas/fuel crossover, high cost of materials, and production of harmful wastes [6,15,16]. Hence, the use of complex fluorine chemistry involved in the PFSA PEM fabrication method is not only the reason for the high stability in the oxidative and reductive conditions of these membranes, but also for their high price and environmental hazards [10]. Noteworthy is that the cost of the membrane is an important point because the PEM material is one of the major contributors (along with catalysts in these systems) to the cost of the membrane electrode assembly [17]. These shortcomings and the need for environmentally friendly and cheap power generation systems have initiated a worldwide research effort to develop alternative PEMs for fuel cells.

Today, the alternate membranes’ designs to Nafion^®^ are partially fluorinated membranes, membranes with hydrocarbon polymer matrixes, inorganic–organic hybrid membranes, acid–base complexes, and grafting membranes via irradiation [6,10,16]. Introducing additives in the membrane, i.e., ionic liquids [18,19,20,21] and inorganic compounds [22,23,24,25,26,27] is used to improve mechanical strength, thermal stability, ionic conductivity, water behavior, and other properties of the PEM. A detailed review on the effects of some dopants on PFSA membrane behavior can be found in Ref. [28].

Among this variety of polymers and dopants, partially fluorinated membranes based on the PVDF copolymers attract special attention [29,30,31,32,33,34,35]. Such membranes are cheaper, easy to manufacture and flexible in changing properties [29,30]. By varying the synthesis conditions, different exchange capacity, water content, porosity, and degree of cross linking of such materials can be achieved. Significant improvement of the propertied of the PVDF-based membranes intended for direct methanol fuel cells can be achieved by incorporating sulfonated Fe_3_O_4_@SiO_2_ nanorods [36] or sulfonated graphene oxide [37] into the PVDF structure. In addition, membranes with the PVDF copolymers’ matrix can be suitable not only for PEMFC applications but also in electrodialysis, electro deionization, membrane electrolysis, etc. [31,38,39,40,41,42]. 

This work reports systematic studies on the synthesis and pivotal physicochemical/ion transport properties of a PVDF-based sulfonated proton exchange membrane. The electrochemical attributes of the synthesized fluorinated membranes were compared with those of commercially available PFSA-based PEMs and cation exchange membranes with the sulfonated PVDF matrix designed for electrodialysis application. This comparison allows us to determine the most promising applications of fabricated membranes and outline ways of their further improvement. 

## 2. Results and Discussion

**Figure 1:**
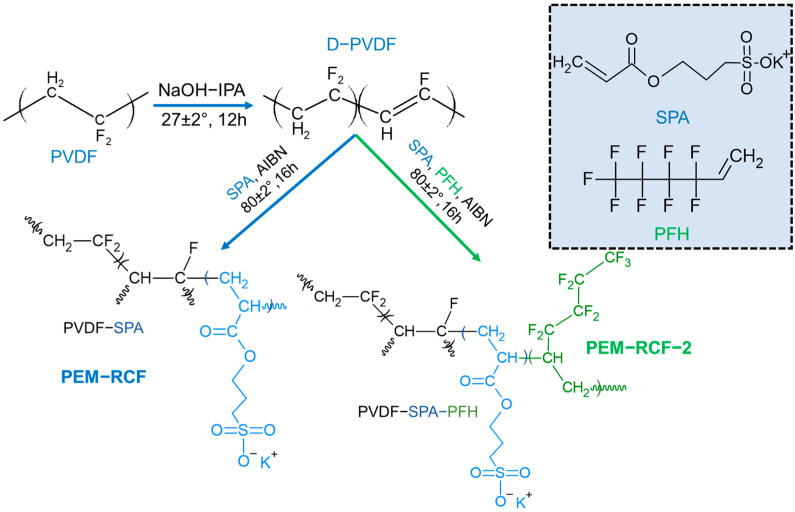
Synthetic route adopted for PEM-RCF and PEM-RCF-2 preparation. Black colour indicates the hydrophobic component (PVDF and PFH), and blue colour indicates the hydrophilic component of the copolymer (SPA). AIBN is azobisisobutyronitrile; IPA is isopropyl alcohol.

In this research, polyvinylidene fluoride-based membranes PEM-RCF and PEM-RCF-2 were synthesized. The chemical modification of PVDF to prepare membranes was achieved as follows: first, dehydrofluorination of PVDF in a basic medium [31] led to a PVDF-containing double bond in the backbone (D-PVDF). Such a double bond could react with 3-sulfopropyl acrylate (SPA) and 1H, 1H, 2H-perfluoro-1-hexene (PFH) under radical initiation to lead to the membranes [43]. Figure 1 illustrates the reaction steps and synthetic strategy adopted for PEMs’ fabrication.

### 2.1. Structural Characteristics of the Membranes under Study 

**Figure 2:**
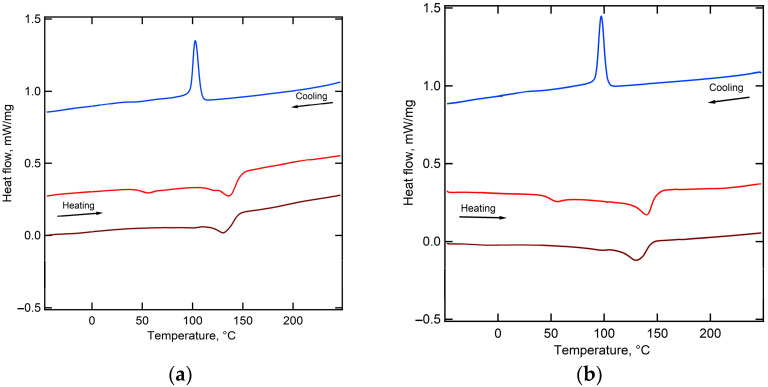
DSC traces of PEM-RCF (**a**) and PEM-RCF-2 (**b**). Red curves represent the first heating cycle, brown curves correspond to the second heating cycle, and blue curves indicate the cooling phase.

The perturbations in melting behavior and crystallinity of functionally modified PVDF via grafting of 3-Sulfopropyl acrylate and 1H, 1H, 2H-Perfluoro-hex-1-ene were studied using the DSC technique. Consequently, the melting of the PVDF phase is observed for PEM-RCF (Figure 2a) and for PEM-RCF-2 (Figure 2b), with an onset temperature of 121 °C and 127 °C, respectively. The reduction in melting temperature in comparison with pure PVDF might be caused by disturbance of the crystalline phase of PVDF by a small fraction of grafted side chains according to Flory’s approach [44]. The enthalpy is 18.2 J/g for PEM-RCF and 17.2 J/g for PEM-RCF-2. Given the melting enthalpy of 104.5 J/g for 100% crystalline PVDF [45], the degree of crystallinity is estimated to be 17% and 16% for PEM-RCF and PEM-RCF-2, respectively.

**Figure 3:**
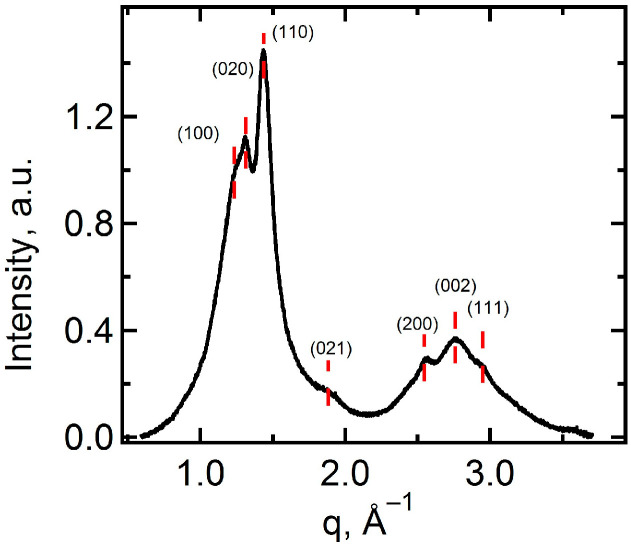
WAXS diffractogram of the PEM-RCF membrane with peak indexation.

**Figure 4:**
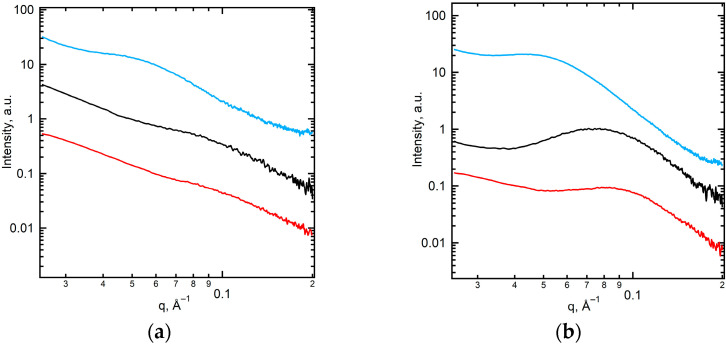
SAXS profiles of PEM-RCF (**a**) and PEM-RCF-2 (**b**) as prepared (red curves), after treatment (black curves), and after recrystallization from the melt (blue curves).

The indexation of the WAXS peaks results in the identification of the α-phase of PVDF [46], as shown in Figure 3. The peaks are broadened due to the small lateral crystal size (5–6 nm). The SAXS curve for the PEM-RCF-2 membrane, as shown by the red line in Figure 4b, exhibits a significant peak *L*_1_ = *Lc* + *La* = 7.9 nm, characteristic of the lamellar stacking in PVDF which includes alternating crystalline (*Lc*) and amorphous (*La*) layers [42,43]. The absence of this peak in PEM-RCF, indicated by the red line in Figure 4a, suggests a less ordered structure, potentially enhancing the membrane’s transport properties since proton transport predominantly occurs through the amorphous regions. Interestingly, after the treatment of PEM-RCF-2 described in Section 3.3, the SAXS peak shifts to smaller angles corresponding to long period *L*_2_ = 9.0 nm because of water absorption into the interlamellar amorphous regions (Figure 4b, black line). The weight fraction of absorbed water WSAXS can be estimated by assuming a typical scenario where the thickness of the crystalline and amorphous layers in lamellar stacks is equal, i.e., *Lc* = *La* = 0.5 *L*_1_. Taking into account the densities of the amorphous and α-crystal phase of PVDF of 1.73 and 1.92 g/cm^3^ [47], respectively, one can estimate *W_SAXS_* as:

(1) $$W_{SAXS}=\frac{\rho_{w}(L_{2}-L_{1})}{\rho_{w}(L_{2}-L_{1})+\rho_{c}L_{c}+\rho_{a}L_{a}}$$

where *ρ_w_*, *ρ_c_* and *ρ_a_* represent the densities of water and the crystalline and the amorphous phase of PVDF, respectively. The water content in PEM-RCF-2 after treatment in sulfuric acid, as calculated from Equation (1), is ca. 7% wt%ht. In contrast, a similar treatment of PEM-RCF does not lead to any noticeable changes in the membrane morphology (Figure 4a, black line). During heating and subsequent crystallization, the lamellar morphology of both membranes becomes more pronounced, i.e., the intensity of the main interference peak, or the long period, increases, while its position shifts to 13.5 and 15.2 nm for PEM-RCF and PEM-RCF-2, respectively (Figure 4a,b, blue lines). Since the recrystallization of the membranes occurs in a dry state, the increase in the long period can be associated with lamellar thickening of α-crystals. This structural change could negatively influence the water uptake and proton diffusion across the amorphous regions, showing a direct correlation between the crystal and supramolecular structures and the transport properties of the membranes under study.

**Figure 5:**
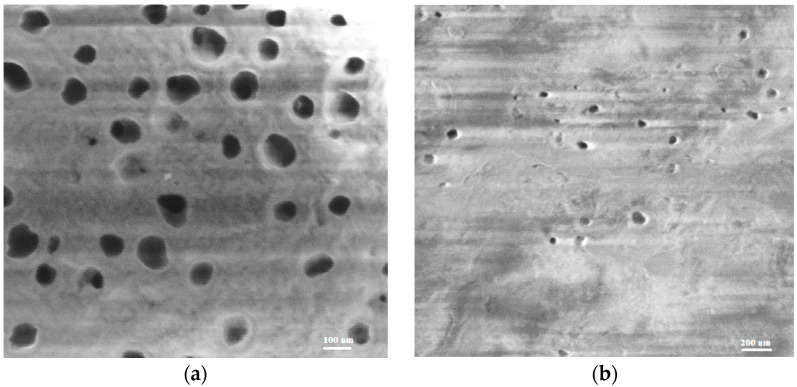
SEM surface images of PEM-RCF (**a**) and PEM-RCF-2 (**b**).

SEM images of both PEM-RCF membranes (Figure 5) reveal a porous structure. As evidenced, PEM-RCF is more porous, with variation in the pore diameters in the range of 56–138 nm and a pore density of 1.4 × 10^9^ cm^−2^. These factors might have a great impact on the transport characteristics. Table 1 provides a summary of the structural information on the membranes.

### 2.2. Structural Characteristics of the Membranes Used for Comparison

The transport properties of ion-exchange membranes (ionic conductivity, permselectivity, and some others) are controlled by their porous structure, including pore size distribution, their direction in space and possible agglomerations, and the physicochemical characteristics of the pore walls [48,49]. In order to obtain an idea of the porous structure of the designed membranes, their transport and electrochemical properties were compared with the corresponding properties of some commercial membranes. 

Two perfluorosulfonated membranes, Nafion^®^ 117 (DuPont Company, Wilmington, DE, USA) and MF-4SK (Plastpolymer, Saint Petersburg, Russia), are chosen as usual references of PEM for the fuel cells. These membranes consist of an aliphatic perfluorinated backbone and ether-linked side chains with sulfonated cation exchange sites. The pore structure is schematized as a system of hydrated clusters with a diameter of about 4 nm, which are connected by channels with 1 nm in diameter and length [50]. The relatively large clusters (the aggregates of fixed sulfo groups with mobile counterions) condition high ionic conductivity, and the presence of narrow channels creates an electrostatic barrier for the anion transport, which ensures high permselectivity of these membranes [51]. The channels ensure the percolation [52] of the membrane, the degree of which can be different with regard to the counterions and co-ions [53]. According to the standard contact porosimetry data [54,55,56], most of the pores contained in these membranes have a radius less than 10 nm. However, there are also pores with a radius of up to 50 nm, but there are no macropores with a radius higher than 100 nm. 

Cation-exchange CJMC-3 and CJMC-4 membranes (ChemJoy Polymer Materials, Hefei, China) are chosen for the comparison, since their ion exchange matrix is based on polyvinylidene fluoride (PVDF) functionalized with sulfonic-$SO_{3}^{-}$ groups and cross-linked with an aromatic agent, similar to the novel membranes under study. Membranes of this type contain nanometer-scale pores. In addition, the crosslinking degree of the CJMC-3 ion-exchange matrix is relatively low; it contains polyester-reinforcing fiber, which causes the presence of macropores localized at the matrix/reinforcement fiber interface [38].

A track-etched polyethylene terephthalate membrane with the provisional name TEM#811 (made by the Flerov Laboratory of Nuclear Reactions, Joint Institute for Nuclear Research, Dubna, Russia) [57,58] is added for the comparison, since it contains through pores with a radius of 20 nm and a pore density of 5 × 10^9^ (estimated by SEM [59]), comparable with the pores of the PEM-RCF membrane (Table 1). The pore walls contain cation-exchange hydroxyl and carboxyl groups within an ion-conducting gel loose layer of thickness of a few nm.

The thicknesses of Nafion^®^ 117, MF-4SK, CJMC-3, and CJMC-4 membranes are relatively close at 230 μm [60], 190–230 μm [61], 185 μm [38], and 128 μm (our data), respectively; while that of TEM#811 is significantly less at 10 μm [59].

### 2.3. Transport Characteristics

Some characteristics of the membranes under study are presented in Table 2.

**Figure 6:**
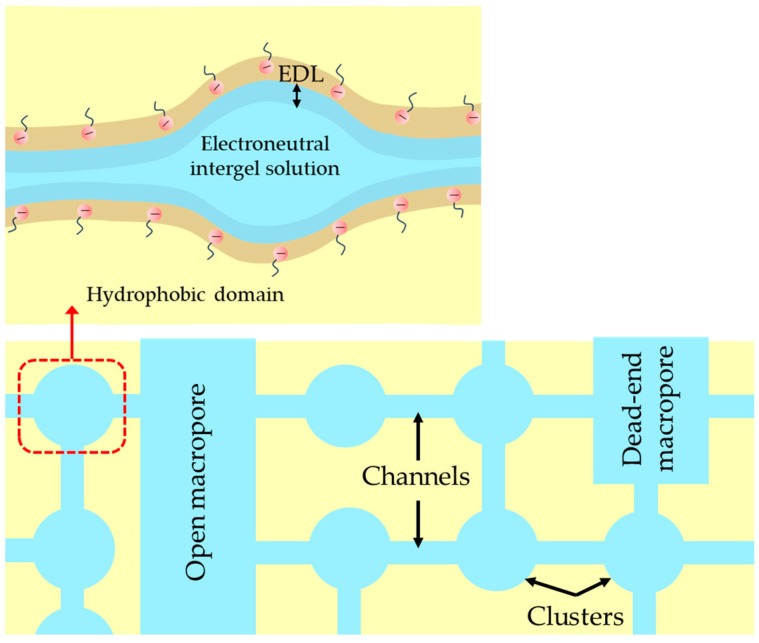
Probable structure of a swollen PEM-RCF membrane. EDL is an electrical double layer.

Comparison of the membranes in Table 2 shows that the PVDF-based membranes (the PEM-RCF one and the CJMC ones) are characterized by a relatively high water content despite the fact that the PEM-RCF and the CJMC-3 display low exchange capacities. Only the PEM-RCF-2 membrane, having an extremely low exchange capacity, is distinguished by a low W value; according to the SAXS result, the water in this sample is localized in the amorphous regions and probably does not form continuous channels. Nevertheless, there is a maximum amount of water per mole of fixed groups for both membranes. The high water content in the manufactured sample PEM-RCF can be partly caused by a fairly high density of macropores and their large radius. However, even when assuming that these pores are through, a simple evaluation shows that their volume fraction is insignificant and amounts to about 8%. It is more likely that the high water content is due to the presence of hydrophilic fragments specially introduced into the polymer matrix, which contain oxygen and sulfonate groups (Figure 1). These hydrophilic fragments should contribute to the formation of a system of nanopores similar to that in Nafion^®^ and other PEM [67,68], which contains ionic clusters and channels (Figure 6); the latter are filled with an aqueous electrolyte solution. The rather high hydrophilicity of the synthesized polymer is also indicated by the smallest contact angle of the PEM-RCF surface (40°), among other studied membranes (Table 2). Noteworthy is that the contact angle of the PVDF-based CJMC-3 membrane is also small despite its low exchange capacity. This small value might be due to the presence of open extended macropores on the surface of the CJMC-3 membrane [38].

**Figure 7:**
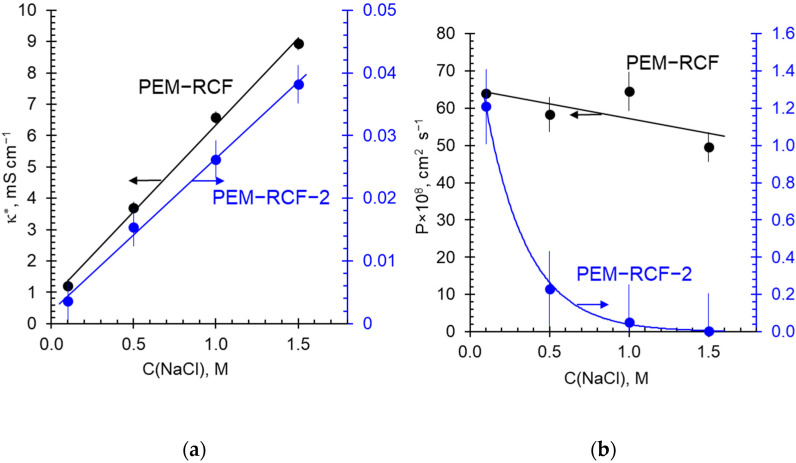
Concentration dependences of the membrane conductivity, κ*, (**a**) and integral diffusion permeability coefficient, *P*, (**b**) of the PEM-RCF and PEM-RCF-2 membranes in NaCl solutions. The 95% confidence interval of the measurement is marked by a vertical line. The solid lines are guides for the eye.

**Figure 8:**
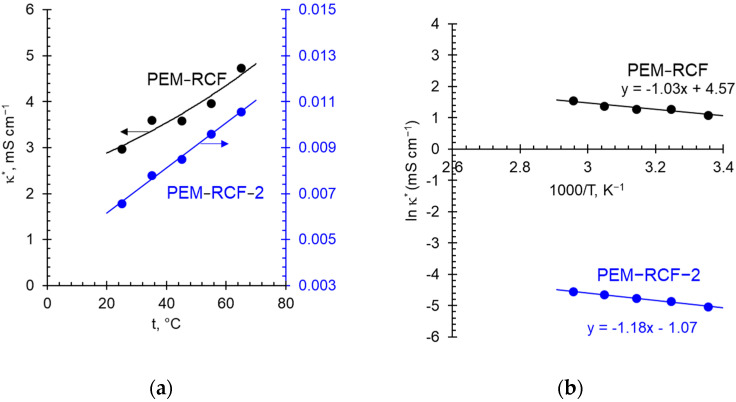
Dependence of the membrane conductivity in 0.05 M HCl solution, κ*, as a function of temperature (**a**) and in the Arrhenius coordinates (**b**). The solid lines are guides for the eye. The equations correspond to a linear regression of the experimental data. Data for Nafion® 117 membrane are taken from Ref. [69].

Figure 7 exhibits the concentration dependences of the conductivity and integral diffusion permeability coefficient of the synthesized membranes in sodium chloride solutions. Figure 8 presents the temperature dependences of the conductivity of these membranes in a 0.05 M HCl solution.

The conductivity of both synthesized membranes increases almost linearly with the increasing concentration of the external NaCl solution. The electrical conductivity of the PEM-RCF sample turns out to be an order of magnitude higher than that of the PEM-RCF-2 sample, which evidently is due to the low exchange capacity of the latter. The temperature dependences of the membrane conductivity presented in Arrhenius coordinates have a classical linear shape [69,70,71,72] (Figure 8).

The diffusion permeability, *P*, of both membranes decreases as the NaCl concentration of the external solution increases. Apparently, this decrease is due to the fact that with the increasing external solution concentration, the membranes lose water. The latter is caused by a decrease in the difference in osmotic pressure between the internal and external solutions (the “swelling pressure” [73]), which leads to shrinkage of the membrane matrix and narrowing of the pores [73]. The decrease in *P* in the case of PEM-RCF-2 is very strong; at the NaCl concentration of 1.5 M, the value of *P* of this membrane is 50 times lower than that of the PEM-RCF membrane. It seems that the channel conductance regarding the co-ion (Cl^−^) becomes so low that the co-ion percolation of the PEM-RCF-2 membrane is lost at a certain external solution concentration.

### 2.4. Comparison of Transport and Electrochemical Characteristics of Synthesized and Commercial Membranes

The activation energy of conductivity, found according to Equation (9) (Section 3.3) from the lnκ*−1/T dependences (Figure 8b), is equal to 8.6 kJ mol^−1^ (for the PEM-RCF membrane) and 9.7 kJ mol^−1^ (for the PEM-RCF-2 membrane), which is close to that of 9.4 kJ mol^−1^ for Nafion^®^ 117 as obtained by Yurova et al. [69]. Note that the activation energy of the proton transfer in aqueous solutions of acids, including the case where such a solution is in a macropore, is about 5 kJ/mol [74]. It is known that the activation energy for conductivity is equal to the sum of the activation energy for proton migration and half the energy for the formation of defects in the membrane. It decreases in the presence of large pores, in which the proton transport occurs via the Grotthuss mechanism [75,76]. On the contrary, the activation energy of conductivity increases if the transferred protons are located at a large distance from each other (low concentration of fixed groups) in pores of a small radius. A similar effect is observed also if the pores are isolated [77]. Our data indicate that the sizes of the pores, which are formed in the synthesized membranes in conditions of a relatively dilute bathing solution (0.05 M HCl), are comparable to the pore sizes formed in the Nafion^®^-117 membrane.

The conductivity of the PEM-RCF membrane in the H^+^-form is about three times higher than that in Na^+^-form (Figure 7 and Figure 8). At 25 °C and when the bathing solution concentration is 0.05 M, in the first case, the PEM-RCF conductivity is about 3.0 mS cm^−1^, while in the second case, 1.0 mS cm^−1^. A similar ratio is observed for Nafion® membranes. For example, at 25 °C and close concentrations, the conductivity of a Nafion® 425 membrane is 16 mS cm^−1^ and 3.6 mS cm^−1^ in the H^+^- and the Na^+^-form, respectively [78]. For Nafion® 117, this ratio is slightly higher: 51 mS cm^−1^ [69] and 9.3 mS cm^−1^ [79] in 0.05 M HCl and 0.05 M NaCl solutions, respectively. The higher conductivity of cation-exchange membranes in the H^+^-form in comparison with the Na^+^-form is due to the higher mobility of H^+^ ions. However, the increase in membrane conductivity (3-5 times) is lower than the increase in ion mobility in the free solution: the mobility/diffusion coefficient of H^+^ in aqueous solutions is about seven times higher than that of Na^+^. In the literature, two mechanisms of proton transport in cation-exchange membranes are discussed [48,49]: vehicular motion, which is the movement of the center of mass of the proton in an aqueous environment, and the Grotthuss mechanism, which is proton shuttling through the hydrogen-bonded network. The contribution of the Grotthuss shuttling, which determines the high mobility of H^+^ ions, increases with the increasing water content in a cation-exchange membrane.

**Conductivity and diffusion permeability**

**Figure 9:**
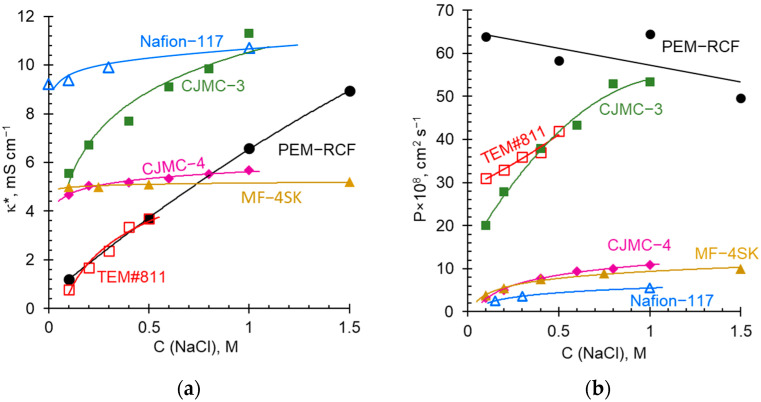
Concentration dependences of the electrical conductivity κ* (**a**) and integral diffusion permeability coefficient *P* (**b**) of the membranes: PEM-RCF, Nafion® 117 [79], MF-4SK [63], TEM#811 [62], CJMC-3 [38], and CJMC-4 in NaCl solutions; if the data were taken from a reference, it is indicated. The solid lines are guides for the eye.

**Figure 10:**
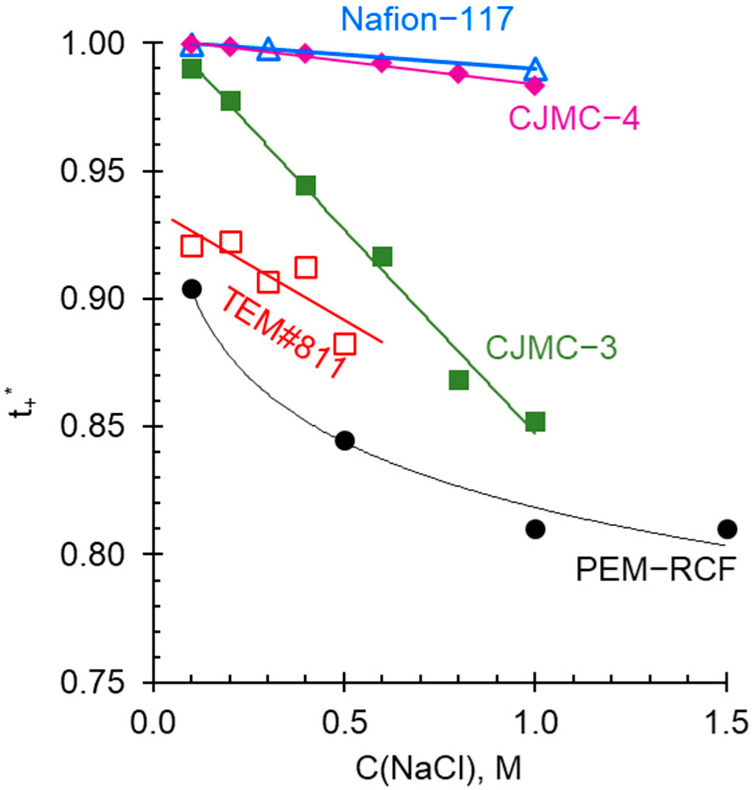
Concentration dependence of the counterion transport number t_−_* in the synthesized membrane PEM-RCF, Nafion® 117 [79], MF-4SK [63], TEM#811 [62], CJMC-3 [38], and CJMC-4; if the data were taken from a reference, it is indicated. The solid lines are guides for the eye.

Figure 9 shows the concentration dependences of conductivity and integral diffusion permeability of the PEM-RCF sample as well as the commercial membranes selected for comparison. Figure 10 presents the counterion transport numbers in these membranes. We measured the characteristics of the CJMC-4 in this study; the data for other commercial membranes are taken from the literature: Nafion® 117 [79], MF-4SK [63], TEM#811 track-etched membrane [62], and CJMC-3 [38].

The shape of the concentration dependences of the conductivity of the membranes presented in Figure 9 can be explained using the microheterogeneous model [80]. This model considers the membrane as a microheterogeneous swollen medium, which consists of two phases: (i) The intergel phase is an electrically neutral solution, which is located in the center of the macro- and mesopores. It is assumed that this solution is identical to the external equilibrium solution; (ii) the gel phase combines all the other components of the membrane: it is a microporous medium containing the polymer chains with fixed groups (sulfonate groups in our case). Additionally, the charge of the fixed groups is compensated by a charged aqueous solution filling the hydrophilic domains of the membrane. There are mobile counterions (carrying a charge of the same sign as the fixed ions) and, in smaller quantities, co-ions (carrying the opposite sign of charge). The gel phase also involves an inert filler (if any) and reinforcing fabric (if any). The conductivity of the gel phase, $\bar{\kappa }$, to a first approximation is considered a constant value, which depends on the counterion diffusion coefficient, $\bar{D_{i}}$, in this phase, and on its exchange capacity, $\bar{Q}$:

(2) $$\bar{\kappa }=\frac{z_{i}{\bar{D}}_{i}\bar{Q}F^{2}}{RT}$$

The value of $\bar{Q}$ is related to the exchange capacity of the membrane *Q*:

(3) $$\bar{Q}=Q/f_{1}$$

where *f*_1_ is the volume fraction of the gel phase. The volume fraction of the intergel phase *f_2_ =* 1 *− f*_1_.

In the case where (1) the solution concentration is close to the concentration of the iso-conductance point (where the conductivities of the membrane and solution are equal), and (2) the membrane contains approximately an equal number of pores, which are connected between them in parallel and in series (regarding the direction of the current flow), the conductivity of the membrane ($\kappa^{*}$), that of the gel phase ($\bar{\kappa }$) and the electrically neutral solution in the intergel spaces (*κ*), are related to each other by the following equation:

(4) $$\kappa^{*}={\bar{\kappa }}^{f_{1}}\kappa^{f_{2}}$$

Using Equation (4), it is not difficult to find the values of *f*_2_ and *f*_1_, as well as $\bar{\kappa }$, if the experimental results are presented in the $\mathrm{lg}\kappa^{*}$ vs. lgκ (or $\mathrm{lg}\kappa^{*}$ vs. lgC) coordinates, since $\mathrm{lg}\kappa^{*}=f_{1}\mathrm{lg}\bar{\kappa }+f_{2}\mathrm{lg}\kappa \approx const+f_{2}\mathrm{lg}C$, when assuming that the electrolyte solution conductivity is proportional to its concentration *C*.

It follows from Equation (4) that in dilute solutions, the main contribution to the membrane conductivity comes from the conductivity of the gel phase. As the concentration of the external solution increases, the conductivity of the intergel space makes an increasing contribution. Moreover, the higher the value of *f*_2_, the more significant this contribution is, which manifests itself in an increase in the slope of the $\mathrm{lg}\kappa^{*}$ vs. lgκ (or $\mathrm{lg}\kappa^{*}$ vs. lgC) dependence. Indeed, in the region of dilute solutions (about 0.1 M), the conductivity of membranes increases in the series TEM#811 ≤ PEM-RCF < CJMC-4 ≈ MF-4-SK < CJMC-3 < Nafion^®^ 117 (Figure 9a), that is, symbatically with the value of the exchange capacity of swollen membranes (Table 2). An increase in the external solution concentration leads to an increase in conductivity of all membranes. The highest growth in conductivity is manifested by the track-etched TEM#811 membrane and the novel PEM-RCF membrane. Apparently, it is due to the extended relatively large open pores, which have these membranes. It is difficult to use the narrow concentration range in the dilute solution region when measuring conductivity, as required by Equation (4). The requirement of an equal number of parallel and serial connections between the pores is also not always met. Therefore, to quantify the rate of increase in conductivity with the increasing concentration, it is convenient to use the apparent volume fraction of the intergel solution [81]:

(5) $$f_{2app}=\frac{d\mathrm{lg}\kappa^{*}}{d\mathrm{lg}C}$$

As Table 2 shows, the *f*_2*app*_ values are the highest in the case of TEM#811 and PEM-RCF having large open pores. In contrast, the perfluorosulfonated membranes do not have large pores, and the values of this parameter is close to 0.1. The CJMC-4 membrane based on PVDF, similarly, has a small *f*_2*app*_ value. However, its homologue, the CJMC-3 membrane, has an increased *f*_2*app*_ value, which should be due to the presence of macropores between the polyester-reinforcing fiber and the membrane matrix.

If the conductivity of ion-exchange membranes (IEMs) is controlled by counterions, then the diffusion permeability is controlled by co-ions. When an electrolyte diffuses through a membrane, counterions cannot leave the membrane without being accompanied by co-ions due to the electroneutrality conditions. The co-ion concentration is low in ion-exchange membranes due to the Donnan exclusion effect: co-ions are electrostatically pushed out by the fixed ions [73]. However, co-ions are excluded only from the gel phase containing the fixed ions. They are not pushed out from the central parts of the macro- and mesopores, where their concentration is the same as in the external solution. The co-ion concentration in the gel phase, under the first approximation, is inversely proportional to the fixed ions concentration, i.e., to the exchange capacity:

(6) $${\bar{c}}_{A}=\frac{K_{D}^{}}{\bar{Q}}C_{}^{2}$$

Equation (6) is obtained from the Donnan equation [73] in the case of the 1:1 electrolyte under the condition that *C* << $\bar{Q}$. The $\bar{Q}$ value determines the degree of electrostatic (Donnan) exclusion of co-ions from the gel phase; a low value of $\bar{Q}$ facilitates the sorption of co-ions; hence, it advances the electrolyte diffusion through the membrane. Another parameter, which controls the membrane diffusion permeability, is *f*_2_. The latter determines the membrane volume fraction, for which the electrostatic exclusion of co-ions is absent.

Indeed, Figure 9b shows that among the studied membranes, two groups can be distinguished: a group (PEM-RCF, TEM#811 and CJMC-3 membranes) with low $\bar{Q}$ (*Q*) and high *f*_2_ (*f*_2*app*_), which is characterized by elevated values of *P*, and a group (CJMC-4, MF-4SK and Nafion^®^ membranes) with elevated $\bar{Q}$ (*Q*) and low *f*_2_ (*f*_2*app*_), which is endowed with a high diffusion permeability.

With the increasing concentration, the effect of co-ion exclusion weakens, since its driving force is the Donnan potential (the difference in potential in the membrane gel phase and in the equilibrium solution), which is close to the ratio *C*/$\bar{Q}$ [73]. Thus, an increase in the solution concentration should lead to an increase in the membrane diffusion permeability. Indeed, this trend can be seen for all compared membranes, except for the PEM-RCF (Figure 9b). The diffusion permeability of this membrane decreases as the external concentration increases. Thus, an increase in the solution concentration should lead to an increase in the diffusion permeability of the membranes. Indeed, this trend can be seen for all compared membranes, with the exception of the PEM-RCF. As we wrote in Section 3.3, the reason for such a dependence in the case of PEM-RCF is a decrease in the degree of membrane percolation with respect to co-ions. This decrease is due to the loss of water by the membrane upon contact with a concentrated solution: shrinkage of the membrane results in narrowing the pores, which hinders the ion transport, but primarily the co-ion transport.

The reason for this behavior of the PEM-RCF membrane is the low concentration of fixed groups, causing the appearance of singularities in its structure as compared to conventional IEMs (Figure 6). Since the number of fixed ions in a cluster is almost independent of their average concentration in the membrane, a decrease in their concentration primarily leads to an increase in the distance between clusters [82,83]. Thus, the length of channels in the PEM-RCF membrane should be longer and their radius smaller than the corresponding parameters in such membranes as Nafion^®^ 117 and MF-4SK. Dehydration of the membrane causes a decrease in swelling and, consequently, a decrease in cluster size, as well as a decrease in the radius of inter-cluster channels [83]. These structural changes cause a sharp decrease in the diffusion coefficient, especially of co-ions. This phenomenon is due to the fact that along with a decrease in the radius of clusters and channels when contacting with concentrated solutions, there is an increase in the concentration of fixed groups per volume of water absorbed by the membrane [73,84,85]. As shown in [86,87], an increase in the concentration of fixed groups should lead to a stronger electrostatic exclusion of co-ions from the channels, which causes an increase in their resistance against co-ions. The high resistance of some channels may cause them to be completely impermeable to co-ions. The need to search for longer pathways of co-ion transport will lead to a sharp increase in the tortuosity factor, which can also be interpreted as a decrease in the degree of membrane percolation with respect to co-ions. These effects are particularly strong in the case of non-crosslinked polymers, such as the PEM-RCF matrix. Polymer chains of the Nafion^®^ 117 and MF4-SK membrane matrix also do not contain additional crosslinking. However, during the manufacturing process, these membranes are subjected to a special temperature treatment that results in the formation of crystallites from the main chains of the polymer. It gives commercial Nafion^®^ 117 and MF-4SK membranes additional rigidity [88]. Therefore, the influence of external electrolyte concentration on pore size and moisture content is less evident in these membranes. The described phenomenon of membrane shrinkage in concentrated solutions is almost absent in the case of the TEM#811 track membrane, since it is based on hydrophobic material that is not capable of swelling.

**Membrane selectivity**

An important property of IEM is selectivity with respect to counterion transport, which can be characterized by (“true”) ionic transport numbers, $t_{i}^{*}$ (Figure 10). According to Equation (12) (Section 3.3), in the first approximation when the counterion transport numbers are close to one, the $t_{i}^{*}$ is proportional to the membrane conductivity and inversely proportional to its diffusion permeability.

Nafion^®^ 117 and CJMC-4 membranes, which do not contain macropores and apparently have a well-balanced combination of hydrophobic and hydrophilic components of the ion exchange matrix, as well as a sufficiently high exchange capacity, are characterized by a very high selectivity. The combination of these properties ensures only a slight decrease in the selectivity of these membranes with the increasing electrolyte concentration in the external solution. The values of counterion transport numbers in the TEM#811 track membrane and the synthesized PEM-RCF sample are more sensitive to the external electrolyte concentration. However, in the case of dilute (≤0.1 M) solutions, $t_{i}^{*}$ exceeds 0.9. The transport numbers of the cations should become even greater, approaching one, with further dilution of the solution. Since PEMs in fuel cells are in contact with the water, the selectivity of PEM-RCF should not be a barrier to its use in this application.

### 2.5. Electrochemical Characteristics

**Figure 11:**
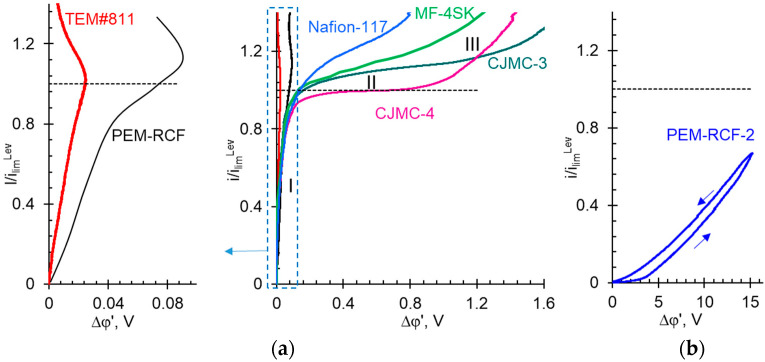
Current-voltage characteristics of PEM-RCF sample and commercial ion exchange membranes (the membrane names are indicated near the curves) (**a**) as well as PEM-RCF-2 sample (**b**). The CVCs of PEM-RCF, PEM-RCF-2, CJMC-3, and CJMC-4 membranes are measured in 0.05 M HCl solutions, as well as that of Nafion^®^ 117, MF-4SK, and TEM#811 in 0.02 M NaCl solutions. The black dashed line corresponds to *i = i*_lim_^Lev^. The blue arrows indicate the direction of current sweep during the CVC measurement of the PEM-RCF-2 membrane.

Figure 11 shows the current-voltage curves (CVC) of the synthesized samples as well as commercial ion exchange membranes in a 0.05 M HCl solution or in a 0.02 M NaCl solution. The values of current densities are normalized by theoretical limiting current density *i*_lim_^Lev^ to offset differences caused by unequal values of diffusion coefficients of H^+^ and Na^+^ cations in the solution. For the same reasons, the ohmic resistance of the space between the Luggin measuring capillaries containing the membrane and the unpolarized solution was subtracted from the measured potential differences.

The low ion transport characteristics of the PEM-RCF-2 sample cause a specific shape of its current-voltage curve, which resembles the CVCs of bipolar membranes [89]. The current-voltage curves of all other membranes have a shape characteristic of others IEMs obtained in many studies [38,90,91,92,93,94,95,96,97]. Initial linear region I of the CVCs shown in Figure 11a corresponds to the formation of diffusion layers near the IEM surface, one of which is depleted in the ions and the other is enriched. At *i <* 0.9*i*_lim_^Lev^, electric current is transported via electrodiffusion: ions move under the influence of the electric potential gradient and the concentration gradient. The sloping plateau (region II of the CVC) occurs when there is a deficit of electric charge carriers in the depleted solution layer at the membrane boundary. The electrolyte concentration near the membrane surface approaches zero, which causes a rapid resistance increase. A very small increase in the current leads to a significant increase in the potential difference, which results in the appearance of a sloping plateau on the CVC. Further rapid growth of the current (region III in Figure 11a) is due to the appearance of new mechanisms of the charge transport. These include (1) electroconvection: the transfer of a volume containing a space electric charge under the action of a potential gradient [98], and (2) “water splitting”: the generation of H^+^ and OH^−^ ions due to the dissociation of water molecules with the catalytic participation of functional groups of the IEM [99]. The slope and length of region II are controlled by the rate of these two charge transfer mechanisms, electroconvection, and water splitting. There is a critical value of the potential drop (different for each membrane) which corresponds to the onset of intensive electroconvection and/or water splitting.

The intersection of the tangents drawn to regions I and II of the CVC gives the value of the experimental limiting current, *i*_lim_^exp^. As can be seen from Figure 11, the *i*_lim_^exp^ value for the membranes presented in Figure 11a agrees reasonably well with the theoretical estimation of theoretical limiting current density *i*_lim_^Lev^ using Equation (14) (Section 3.3).

The CVC of the PEM-RCF membrane has the same characteristic regions as those of the other IEMs. The difference is that region II is more diffuse, its slope is much larger, and its length is much shorter than that of the other membranes (excluding TEM#811). Only in the case of PEM-RCF and TEM#811, a negative slope of region III is noted. This slope corresponds to the negative differential resistance of the membrane system: at a small increase in current, there is a decrease, rather than an increase, in the potential drop. Such an effect could be caused by the electroosmotic (EO) transport of the solute via open or extended solution pores. The theoretical possibility of sufficiently intense electroosmosis via TEM#811 pores was discussed in our recent publication [100]. EO transport via the membrane pores is caused by the action of an external electric force on the space charge localized in the double electric field at the surface of the pore walls. The rate of EO transport increases with the increasing potential drop. EO flow via the membrane pores induces a “fresh”, more concentrated solution from the bulk solution into the depleted diffusion layer. This “fresh” solution leads to a decrease in the resistance of the membrane system and the negative diffusion resistance. It is possible that PEM-RCF has a small number of through pores, which makes the effect described above possible.

If the concentration of fixed sulfonate groups of the PEM-RCF sample is increased, a quantitative match of its CVC with the corresponding curves obtained for PEM and IEMs can be expected. Improvement of the electrochemical properties of this sample can be achieved by increasing the number of side chains with sulfonate groups during the synthesis process or by filling its macropores with an ionic liquid or solid polyelectrolyte, as demonstrated in previous studies [101,102,103,104].

## 3. Materials and Methods

### 3.1. Chemicals

In this work, distilled water (electrical conductivity of 3 ± 0.5 μS cm^−1^; pH = 5.5 ± 0.2; 25 °C), solid NaCl of the analytical grade (JSC Vecton, Saint Petersburg, Russia), and liquid standards HCl and NaOH (JSC Lenreaktiv, Saint Petersburg, Russia) were used to prepare the solutions. Polyvinylidene fluoride, PVDF (Solef^®^, M_n_ = ~230,000 and melt flow index = 6–8 g per 10 min; temperature = 230 °C), was purchased from Solvay S.A. (Brussels, Belgium) and dried at 45 °C before chemical modification. The 3-sulfopropyl acrylate (hydrophilic monomer), 1H, 1H, 2H-perfluoro-1-hexene (fluorinated spacer) and Azobisisobutyronitrile (AIBN, radical initiator) were supplied from Sigma Aldrich (St. Louis, MO, USA). Solvents (as dimethyl acetamide, DMAc) for membrane casting were procured from Qualigens Chemical Ltd (Mumbai, India)., while common reagents like iso-propyl alcohol (IPA), sodium hydroxide, and sodium chloride were obtained from B. Hiten chemical distributor. All reagents were used without procession (Except AIBN, which was recrystallized in ethanol prior to copolymer synthesis).

### 3.2. Membrane Preparation: Synthesis of Polyvinylidene Fluoride Copolymer

The formation of the unsaturated site in PVDF is a desirable strategy for the copolymerization of monomers viz. 3-sulfopropyl acrylate potassium salt (SPA) and 1H, 1H, 2H-Perfluoro-1-hexene (PFH) and to synthesize the desired copolymers.

(a) Dehydrofluorination of polyvinylidene fluoride (PVDF)

The dehydrofluorination of polyvinylidene fluoride (PVDF) was carried out using the previous reported method [43]. In brief, a 10 *w*/*v* % of vacuum-dried PVDF powder was prepared in a DMAc solvent. Further, the homogenous solution of PVDF was then subjected to dehydrofluorination using a 0.5 M NaOH/isopropyl alcohol (IPA) reagent at 27 ± 2 °C for 12 h. Finally, the dehydrofluorination reaction was quenched by precipitating the brown-colored polymer from cold water. To eliminate the interference of any trapped solvent, the dehydrofluorinated PVDF was washed multiple times until free from the bound DMAc, and the modified PVDF was dried in vacuum at 75 °C for 24 h.

(b) Synthesis of polyvinylidene fluoride-co-(3-sulfopropyl acrylate) and polyvinylidene fluoride-co-(3-sulfopropyl acrylate)-co-perfluoro-1-hexene copolymer

Simple one-pot free radical polymerization was adopted to synthesize the copolymer of dehydrofluorinated PVDF and 3-sulfopropyl acrylate potassium salt akin Yadav et al. [105]. For the synthesis of polyvinylidene fluoride-co-(3-sulfopropyl acrylate), a 15 *w*/*v* % polymer solution of dehydrofluorinated PVDF was prepared at 45 ± 2 °C in an N_2_ atmosphere. To this solution, 10 *w*/*v* % of 3-sulfopropyl acrylate potassium salt was added and allowed to mix homogenously. Thereafter, the temperature of the reaction mixture was set to 80 ± 2 °C and allowed to further dissolve completely, and Azobisisobutyronitrile (radical initiator) was added at 2 mol % of the monomer concentration in the final reaction mixture. The obtained solution was continued to react for 16 h and the colour changes from pale brown to off white. Finally, the reaction was quenched and allowed to slow dry in a vacuum oven for 24 h at 60 °C to yield the desired polyvinylidene fluoride-co-(3-sulfopropyl acrylate) copolymer. Furthermore, to understand the influence of the fluorinated spacer, the copolymer was finetuned and modified. Akin to the procedures adopted for polyvinylidene fluoride-co-(3-sulfopropyl acrylate) synthesis, the polyvinylidene fluoride-co-(3-sulfopropyl acrylate)-co-perfluoro-1-hexene copolymer was synthesized. During the addition of monomers, the relative input ratio of 3-sulfopropyl acrylate potassium salt: 1H, 1H, 2H-Perfluoro-1-hexene was 9:1 *w*/*w* %.

Finally, the synthesized copolymers with buff and the cream-coloured solution texture were allowed to dissolve in the dimethylacetamide (DMAc) solvent to prepare a homogenous solution. The obtained copolymer mixture was blade casted to the desired thickness and dried for 24 h to obtain the membrane.

### 3.3. Methods of Membrane Characterization

**The morphology and physical properties** of the membrane were studied via small (SAXS) and wide angle (WAXS) X-ray scattering, scanning electron microscopy, and differential scanning calorimetry.

The **SAXS/WAXS measurements** were carried out on a laboratory Xenocs Xeuss 3.0 system equipped with a GeniX3D copper micro-focus tube operating at 60 kV and 0.59 mA with a 1.5406 Å wavelength. The samples were placed in an evacuated chamber and illuminated with monochromatic X-rays in transmission geometry. The scattered intensity was recorded using a Dectris Eiger2 1M detector implemented in a long vacuum flight tube at two different sample-to-detector distances of 68 mm and 0.7 m. The accessed q values, with |q| = 4πsin(ϴ)/λ, where ϴ is the Bragg angle and λ is the wavelength, cover a range from 0.015 Å^−1^ to 3.0 Å^−1^. The degree of crystallinity of the membranes was estimated from the results of WAXS. The 1D diffraction curve was decomposed in IgorPro v.6.37(WaveMetrics) software. The crystal size was calculated with the Scherrer equation from the 110 peak. To study the influence of water penetration into the hydrophilic domains, SAXS measurements were performed for both dry and swollen membranes. The next protocol was used: boiling in milliQ for 6 h and keeping in 3 M H_2_SO_4_ at 50 °C for 5 h after.

**SEM experiments** were performed with a Zeiss Crossbeam 550. The membrane samples were attached to carbon tape and examined without sputtering at an accelerating voltage of 1 kV.

**DSC experiments** were performed with a Mettler Toledo DSC 3+ instrument. The protocol was the same for both samples: two heating and cooling runs with the temperature range from −50 to 250 °C and rates equal to 10 K/min, annealing at room temperature for one day and repeating the same heating/cooling runs. STARe software was used to obtain the enthalpies of phase transitions.

Prior to the next measurements, all membrane samples were subjected to standard salt treatment in the NaCl solutions [55].

The **contact angles** of the membrane were measured using the sessile drop method as reported earlier [66]. A drop of distilled water was placed on the surface of the swollen sample and the image was captured with an optical microscope. Then the contact angle of the surface was obtained via image processing. The measurements were repeated at least 10 times at different points of the membrane surface and the values were averaged.

The **ion exchange capacity** (*Q*) is determined using the static method [55,106]. For this purpose, protons of fixed -SO_3_H groups were replaced with sodium ions using a 0.1 M NaCl solution. Then the amount of removed H^+^ ions was found via titration with a 0.02 M NaOH solution. *Q* is found as the ratio of the number of moles of the H^+^ ions to the mass of the swollen membrane. Detailed information on this and other physicochemical methods of membrane characterization can be found in the Supplementary Materials.

**Water content** (*W*) of the membranes is found using the gravimetric method. This method consists of drying a swollen wet membrane in the H^+^ form, equilibrated with distilled water, initially of mass *m_wet_*, to an air-dry state, while weighing on an analytical balance to a constant mass value (*m_dry_*). The gravimetric water content is calculated according to the formula:

(7) $$W=\frac{m_{wet}-m_{dry}}{m_{wet}}\times 100\%$$

The ion exchange capacity and water content also provide values for the **membrane hydration number** (*n_m_*, mol H_2_O/mol -SO_3_^−^) [63], which is the average number of water molecules per functional group. The n_m_ is calculated according to the formula [63,65]:

(8) $$n_{m}=\frac{W}{M_{w}Q}$$

where *M_w_* is the molar weight of water (18 g mol^−1^).

The **electrical conductivity** (κ*) of the membrane was determined via the differential method using a clip-like cell [107,108]. The membrane resistance is determined as the difference between the resistances of the space between the electrodes with the solution and the membrane and only with the solution.

The **activation energy** of the membrane conductivity is determined using the tangent of the slope of the temperature dependence of κ* in Arrhenius coordinates [60,70,109]:

(9) $$E_{act}=-\frac{d\ln\kappa^{*}}{d(1/T)}R$$

To obtain the **diffusion permeability** of the membranes, a two-chamber flow cell was used [110]. The membrane separated two compartments: distilled water was circulated through one of them (I), and a NaCl solution of a given concentration, *C_II_*, was pumped through the other (II). The diffusion permeability coefficient, *P*, is determined based on the rate of ion diffusion transport through the membrane under the concentration gradient between the solution II and distilled water:

(10) $$P=\frac{Jd_{mb}}{C_{II}}$$

where *J* is the diffusion flux density, and *d_mb_* is the membrane thickness.

The differential diffusion permeability coefficient, P*, related to the concentration *C_II_* is found when knowing the dependence *P*(*C_II_*) [111]:

(11) $$P^{*}=P\left(1+\frac{d\mathrm{lg}P}{d\mathrm{lg}C_{II}}\right)$$

The **transport number of co-ion** (t_−_*) in the membranes under study is obtained using the experimental values of κ* and P* on the base of the relationship deduced by comparing the Onsager and the Kedem–Katchalsky transport equation systems [111]:

(12) $$t_{-}^{*}=\frac{P^{*}F^{2}C}{2RT\kappa^{*}t_{+app}g}$$

where *F*, *R*, and *T* are the Faraday constant, gas constant, and temperature; *t*_+*app*_ is the apparent counterion transport number; *g* is the activity factor; and *C* is the electrolyte concentration of the bathing solution (in this study, it varied in the range of 0.1–1.5 mol L^−1^). When accepting that $t_{+app}^{}\approx t_{+}^{*}$ and *g* ≈ 1, the following approximate equation usually used for calculating the ion transport numbers in the membrane is obtained [111,112]:

(13) $$t_{+}^{*}=\frac{1}{2}+\sqrt{\frac{1}{4}-\frac{P^{*}F^{2}C}{2RT\kappa^{*}}};\;t_{-}^{*}=1-t_{+}^{*}$$

**Current-voltage characteristics (CVC)** of the membranes were studied in a flow-through four-chamber cell. The scheme of the cell as well as the principle of its operation are described in [38]. The potential drop across a membrane under study was measured using two silver/silver chloride electrodes connected to the Luggin capillaries. Further relevant details are given in Supplementary Materials.

The value of the **theoretical limiting current density** was calculated using the Lévêque equation obtained within the convection–diffusion ion transport model for a flat channel with an ideally selective membrane [113]:

(14) $$i_{\lim}^{Lev}=1.47\frac{FDz_{+}C}{h(1-t_{+})}{\left(\frac{h^{2}V}{LD}\right)}^{1/3}$$

where *D* is the electrolyte diffusion coefficient, *z*_+_ is the counterion charge number, *h* is the intermembrane distance, *t*_+_ is the electromigration transport number of the counterion in the solution, *V* is the average linear flow velocity of the solution, and *L* is the length of the active part of the membrane surface.

All experiments were conducted at a temperature of 25 °C, excluding the measurement of the temperature dependence of membrane conductivity.

As for further characterization of the developed membranes, the membranes should be tested for gas permeation and chemical stability following, for example, the procedure suggested in Ref. [114].

## 4. Conclusions

Using a relatively inexpensive material, an attempt has been made to synthesize a graft copolymer that contains hydrophobic fluorine-containing backbones (polyvinylidene fluoride, PVDF, and Perfluoro-1-hexene, PFH) and hydrophilic side chains (3-Sulfopropyl acrylate, SPA). One of the two synthesized membranes (PEM-RCF) demonstrates properties proper to proton-conducting (Nafion^®^ 117, MF-4SK) and ion-exchange membranes (CJMC-3, CJMC-4). It has a low surface resistance (0.9 Ω cm^2^), characterized by an activation energy of fixed groups (8.6 kJ mol^−1^), close to the activation energy of fixed sulfonate groups of the Nafion^®^ 117 membrane (9.4 kJ mol^−1^), but it is inferior to this and other membranes selected for comparison in selectivity. This disadvantage appears to be caused by the relatively small amount of hydrophilic sulfonate-containing moieties introduced into the copolymer during the synthesis step and the formation of regular lamellar stacks of α-crystals of PVDF. These α-crystals act as a barrier to ion transport. In addition, the ion transport and electrochemical characteristics of PEM-RCF are negatively affected by open-extended macropores formed in the membrane, probably at the casting manufacturing stage. Eventually, the influence of PFH hydrophobic spacers suggest a decrement in the ion migration aptitude and a reduction in the conducting phase since the structure is dense and occupied by an inert copolymer of PVDF and PFH. These shortcomings can be overcome by changing the synthesis conditions; for example, by using high boiling dry solvents and the casting of membranes in a vacuum environment at very low humidity and/or filling the macropores of the synthesized membrane with cationic ionic liquids or a solid polyelectrolyte such as Nafion^®^.

## Figures and Tables

**Table 1:** Structural characteristics of the PEM-RCF and PEM-RCF-2 membranes.

Membrane	Degree of Crystallinity, %		PVDF Phase	Grain Size from XRD, nm	Diameter of Open Extended Pores, nm	Pore Density, Pores/cm^2^	Membrane Thickness, ^1^ d_mb_, µm
	DSC	WAXS					
PEM-RCF	17	18	α	5	56–138	1.4 × 10^9^	29 ± 2
PEM-RCF-2	16	19	α	6	20–58	2.8 × 10^8^	86 ± 2

^1^ In the swollen state in equilibrium with 0.05M HCl solution.

**Table 2:** Some physicochemical characteristics of the membranes.

Membrane	Q, mmol g^−1^ Wet	Water Content, W, g_H_2_O_/g_wet_, %	*n_m_*, mol H_2_O/mol Functional Groups	Area Resistance, R, Ω cm^2^	^7^ *f*_2*app*_	Contact Angle, (wet), Grad
PEM-RCF	**^1^ 0.12 ± 0.02**	**^3^ 26 ± 2**	**^3^ 122**	**^5^ 0.9 ± 0.05**	**0.81**	**40 ± 2**
PEM-RCF-2	**^1^ 0.08 ± 0.01**	**^3^ 7 ± 2**	**^3^ 52**	**^5^ 571 ± 3**	-	-
TEM#811	^1^ 0.064 ± 0.003 [62]	^4^ 6.5 [59]	^4^ 43 [59]	^5^ 0.3 [62]	0.95 [62]	**62 ± 3**
Nafion^®^ 117	^1^ 0.71 [60]	^3^ 14.8 [60]	^3^ 14 [60]	^6^ 0.82 [60]	0.1–0.15 [63]	^8^ 108.1 ± 0.7 [64]
MF-4SK	^2^ 0.58 [63]	^3^ 16.7 [63]	^3^ 16 [63]	-	0.05 [63]	77.5 ± 2.6 [65]
CJMC-3	^1^ 0.63 ± 0.05 [38]	^4^ 30 ± 3 [38]	^4^ 27 [38]	^5^ 2.2 ± 0.3 [38]	0.33 [38]	51 ± 2 [66]
CJMC-4	**^1^ 1.2 ± 0.1**	**^4^ 29 ± 3**	**^4^ 13**	**^6^ 2.5 ± 0.1**	**0.09**	64 ± 1 [66]

^1^—equilibrium exchange capacity (H^+^ was replaced by Na^+^ and titrated); ^2^—total exchange capacity (H^+^ directly titrated by OH^−^); ^3^—in H^+^ form; ^4^—in Na^+^ form; ^5^—in 0.5 M NaCl; ^6^—in 0.2 M NaCl; ^7^—determined from measurements of electrical conductivity in NaCl (Section 2.4); ^8^—in dry state. Values obtained in this study are presented in bold.

## Data Availability

The data presented in this study are available on request from the corresponding author.

## Supplementary Materials

The following supporting information can be downloaded at: https://www.mdpi.com/article/10.3390/ijms25010598/s1.
