# Supplementary material for: Structural Characterization and Physicochemical Properties of Functionally Porous Proton-Exchange Membrane Based on PVDF-SPA Graft Copolymers
Source: Int J Mol Sci. 2024 Jan 2;25(1):598. doi: 10.3390/ijms25010598 (PMC10779367; doi:10.3390/ijms25010598)
Supplement: Supplementary file 1 [file ijms-25-00598-s001.zip › ijms-2742563-supplementary.pdf]

S1. Membrane characterization

A high-precision digital micrometer MKC-25 0.001 was used to control the **membrane thickness** (d_{mb}, µm). The results of 10 measurements performed at various points of the sample under study were averaged to obtain the membrane thickness with an accuracy of 1 µm.

The equilibrium exchange capacity (Q, mmol g^{-1} wet) is determined by the static method. Primarily, membrane samples were transformed into the H^+ form and washed in distilled water. Prepared samples are weighed (m_{wet}), cut into pieces and placed into conical flasks. Then the flasks were filled with a sodium chloride solution with a volume of 20.0 cm^3 with a concentration of 0.1 M to replace H^+ ions by Na^+. Then, the samples were kept for 24 hours in the above mentioned solutions. After that, an aliquot of the solution over the membrane (5.00 cm^3) was taken for titration to determine the concentration of the H^+ ions released into the solution. Titration was performed using EasyPlusTitrators (Mettler Toledo) till the equivalence point at pH=7. The calculation of the membrane exchange capacity, Q, was carried out by the following equation:

$$Q = \frac{4C_{NaOH}V_{NaOH}}{m_{wet}} \quad \text{(S1)}$$

where V_{NaOH} is the volume of the solution, spent on titration (mL), C_{NaOH} = 0,02 M, m_{wet} is the mass of swollen sample (g).

Water content (W, %) of the membranes is determined by the gravimetric method. For this purpose, the sample in the H^+ form, equilibrated with distilled water, was weighed in air-dry state on an analytical balance (m_{wet}). Then it was placed in MB24 Ohaus moisture analyzer (Ohaus Europe GmbH, Switzerland). The drying process was carried out at a temperature of 100 °C to constant weight of the sample (m_{dry}). Thus, the water content was defined according to the formula:

$$W = \frac{m_{wet} - m_{dry}}{m_{wet}} \times 100\% \quad \text{(S2)}$$

The electrical conductivity of the membrane (κ^*) was determined using a clip-like cell with 1 cm^2 Pt/Pt electrodes and an immittance meter AKIP 6104 (B + K Precision Taiwan, Inc., New Taipei City, Taiwan) at an alternating current frequency of 1 kHz. The cell scheme, the methodology for conducting the experiment and processing the obtained data are described in detail in [Lteif, R. et al. Eur. Polym. 1999; Karpenko et al. Russ. J. Electrochem 2001]. For the study of temperature dependence, the clip cell was placed in a thermostat LOIP LT-100 (JSC LOIP, Russia) and membrane conductivity was measured in a 0.05 M HCl solution in a range 25-65°C (±2°C). The value of κ^* was determined by the equation:

$$\kappa^* = \frac{d_{mb}}{R_{m+s} - R_s} \quad \text{(S3)}$$

where R_{m+s} is the resistance of membrane and solution; R_s is the resistance of solution; and d_{mb} is the membrane thickness in the solution of given concentration.

The methodology of the procedure for obtaining **diffusion permeability** is detailed in [Shutkina, E.A. et al Condens. Matter Interphases 2015]. The membrane separated two compartments: distilled water was circulated through one of them (I), and a NaCl solution of a given concentration was pumped through the other (II). During the experiment, ions passing through the membrane increase the electrical conductivity of distilled water, which is registered by the conductivity sensor by means of an Ekspert-002 conductometer (Ekoniks-Ekspert, Russia). Before the experiments, the sample was equilibrated with each concentration of electrolyte in the study. The integral diffusion permeability coefficient is determined according to the equation:

$$P = \frac{V_I d_{mb}}{SC_{II}} \frac{dC_I}{dt} \tag{S4}$$

where V_I is volume of the distilled water (100 mL); S is membrane working area (6,25 cm^2); C_{II} is concentration of NaCl (0.1 – 1.5 M); $\frac{dC_I}{dt}$ is the concentration growth rate in the circuit with distilled water.

Current–voltage characteristics of the membranes were studied in a flow-type four-chamber cell. The potential drop across the membrane under study was determined using two silver/silver chloride electrodes connected to Luggin capillaries. The tips of the capillaries are at 2.5 mm from the membrane surface. The potential drop was measured using the Keithley 2010 multimeter (Keithley Instruments, USA); the current was supplied to the cell by the Keithley 2200-60-2 power supply (Keithley Instruments). The parameters of the chambers of the cell were as follows: the intermembrane distance of 6.58 mm and the working surface area of 4.3 cm^2.The linear flow rate was 0.36 cm s^{-1} during the experiment. The presence of slit-like parallel channels in the input and output devices of the cell provided a laminar flow of the solution of the electrolyte. The membrane under study was separated from the anode and cathode by auxiliary MA-41 and MK-40 ion-exchange membranes, respectively. The CVCs were measured in the galvanodynamic mode with a current sweep speed of 0.05 mA s^{-1}. The CVC characteristics were studied in solution of 0.05 M HCl (pH =1.44±0.03).
